# Point Cloud Classification and Segmentation Network Based on Adaptive Feature Extraction

**DOI:** 10.3390/s26123689

**Published:** 2026-06-10

**Authors:** Chengzhi Deng, Huaipei Wang, Zhaoming Wu, Xiaowei Sun, Shaoquan Zhang, Shengqian Wang

**Affiliations:** 1 School of Computer Science and Software, Zhaoqing University, Zhaoqing 526061, China; 2 Key Laboratory of Smart Water Conservancy in Jiangxi Province, Jiangxi University of Water Resources and Electric Power, Nanchang 330099, China; dengchengzhi@126.com (H.W.); wzm@nit.edu.cn (Z.W.); 2013994451@nit.edu.cn (X.S.); dengcz@juwp.edu.cn (S.Z.); 2008994045@nit.edu.cn (S.W.)

**Keywords:** point cloud classification, point cloud segmentation, adaptive feature extraction, Hadamard product, inverted residual MLP, lightweight network

## Abstract

Point cloud classification and segmentation are key technologies for 3D perception and scene understanding, whose accuracy and efficiency directly affect the performance of high-level applications such as 3D modeling, object recognition, and intelligent interaction. Existing methods still exhibit obvious deficiencies in local feature representation, computational efficiency, and scene applicability. To address these issues, this paper proposes a lightweight point cloud classification and segmentation network based on adaptive feature extraction, referred to as AFE-PointNet. Firstly, an element-wise weighting set abstraction module based on the Hadamard product is designed. It leverages geometric topology learning to achieve adaptive feature enhancement, effectively improving the representation capability of local geometric structures. Meanwhile, a cascaded structure of feature aggregation and an inverted residual multi-layer perceptron (InvResMLP) is adopted for deep feature mining to achieve high-accuracy and high-efficiency point cloud classification and segmentation. Experimental results show that AFE-PointNet achieves an overall accuracy (OA) of 93.6% on the ModelNet40 dataset and 84.5% on the ScanObjectNN dataset, and attains a class mean intersection over union (Cls.mIoU) of 83.6% on the ShapeNetPart part segmentation dataset, yielding significant performance improvements over the PointNet++ model. The proposed adaptive feature enhancement and lightweight deep mining strategies effectively improve point cloud representation capability, providing a high-precision and efficient solution for 3D vision tasks.

## 1. Introduction

Point clouds are a data format that directly reflect three-dimensional spatial structures and can accurately describe the geometric features of objects. They have been widely used in various fields such as robot navigation [1], autonomous driving [2], and virtual reality [3]. However, point cloud data exhibit characteristics such as disorder, unstructured distribution, and non-uniform density, which make their processing fundamentally different from traditional two-dimensional data. Therefore, learning how to effectively understand 3D scenes from point clouds has become a critical issue. Point cloud classification and segmentation [4,5], as core technologies for point cloud processing and understanding, can extract structured information from unordered point clouds, thereby supporting object recognition, localization, and reconstruction tasks. Unlike 2D images, point cloud data do not possess regular grid topology and are typically represented as unordered 3D point sets. Meanwhile, the inherent sparsity, high dimensionality, and uneven sampling density of point clouds further increase the difficulty of classification and segmentation tasks. Therefore, designing dedicated deep learning models tailored to these characteristics has become a core research direction in the field of point cloud classification and segmentation.

Before the rise of deep learning, point cloud processing relied heavily on handcrafted features [6,7]. Such methods often suffer from limited generalization ability and restricted both accuracy and efficiency in classification and segmentation tasks. In recent years, with the rapid development of deep learning, researchers have achieved multi-level breakthroughs ranging from object classification to part segmentation by converting raw point clouds into structured representations (e.g., voxels or multi-view projections) and extracting features using mature convolutional neural networks [8,9]. Although such methods have attained preliminary success, they easily cause geometric distortion during transformation and incur prohibitive computational costs due to redundant projection operations [10]. In contrast, PointNet [11] pioneered the direct processing of point clouds using multi-layer perceptions (MLPs) and symmetric functions (e.g., max pooling) to extract global features. However, PointNet only focuses on global representation and fails to effectively model local structures. Although PointNet++ [12] alleviates the shortage of local feature learning to a certain extent, it still suffers from insufficient geometric information extraction, difficulty in preserving topological relationships, and high computational complexity due to limitations in its feature encoding mechanism and network architecture.

To address the aforementioned limitations of PointNet++, this paper proposes an adaptive feature extraction network for point cloud classification and segmentation. Built upon the classic PointNet++ baseline, the presented model integrates an element-wise weighted set abstraction module with a cascaded feature aggregation structure and inverted residual MLPs to enable efficient, adaptive feature learning. This elaborate design substantially enhances the overall point cloud representation capability while effectively lowering computational overhead. Extensive experimental results on three public point cloud datasets demonstrate that the proposed method achieves superior accuracy for both point cloud classification and segmentation tasks.

The main contributions of this paper are summarized as follows:

1. A lightweight point cloud classification and segmentation network (AFE-PointNet) is proposed. Based on an enhanced PointNet++ framework, the network integrates an element-wise weighted set abstraction module and an InvResMLP cascade, achieving a favorable balance between high accuracy and low computational overhead.
2. An adaptive local feature extraction mechanism is developed. Using a Hadamard product-based set abstraction module, the model learns geometric topology adaptively via relative coordinate normalization and channel-wise standardization, enhancing robustness to translation, scaling, and uneven density.
3. An efficient feature aggregation and mining strategy is implemented. The InvResMLP module features an inverted bottleneck structure and residual connections, expanding feature dimensions while ensuring training stability. When cascaded with the set abstraction module, it enables fast convergence and reduced overfitting.
4. State-of-the-art performance is achieved on multiple benchmark datasets. AFE-PointNet attains 93.6% OA on ModelNet40 and 84.5% OA on ScanObjectNN, outperforming PointNet++ by 1.7 and 10.8 percentage points, respectively. On ShapeNetPart, it achieves 86.7% Ins.mIoU, confirming its robust and efficient point cloud understanding.

The rest of the paper is organized as follows: Section 2 reviews the literature on point cloud classification and segmentation. Section 3 details the proposed AFE-PointNet classification and segmentation networks. Section 4 introduces the experimental setup and discusses the results. Section 5 concludes the paper.

## 2. Related Works

Point clouds are inherently unstructured and irregular, leading to high computational and memory costs during processing. A common strategy for point classification and segmentation is to convert point clouds into more regular representations. Voxel-based methods regularize point clouds by dividing 3D space into uniform grids, which helps reduce the impact of irregular distributions. Representative early works include VoxelNet [13] and 3D ShapeNets [14]. To improve storage efficiency and scalability, many later methods replace fixed-resolution voxels with tree-based structures. For example, OctNet [15] employs unbalanced octrees to exploit sparsity in 3D data, while Kd-Net [16] uses KD-tree indexing for structured learning. OctFormer [17] further combines irregular window partitioning with octree structures to accelerate Transformer-based point cloud processing. PVT [18] extracts coarse-grained features from non-empty voxels to avoid redundant computations caused by disordered point arrangements, and CCMNet [19] fuses coarse-, medium- and fine-grained features and utilizes complementary configurations of voxel scale and neighborhood sphere scale to improve performances. Another typical indirect approach is to project point clouds onto multiple 2D views, which allows leveraging mature 2D computer vision techniques. Classical methods include MVCNN [8], View-GCN [9], and GVCNN [20]. However, early multi-view frameworks treat all views equally and cannot emphasize informative viewpoints. To address this issue, MVTN [21] uses a differentiable transformation module to learn optimal viewpoints, and Voint Cloud further unifies multi-view features with native point features. MVACPN [22] reduces information loss during feature encoding and dimensionality reduction using an attention–convolution hybrid pooling mechanism.

Although converting point clouds into intermediate representations alleviates irregularity, it inevitably loses detailed geometric information. Direct methods avoid format transformation and operate directly on raw point sets, making them the dominant paradigm in modern point cloud analysis. While CNNs achieve remarkable performance on grid data, their rigid structure is incompatible with unordered and irregular point clouds. To overcome this limitation, PCNN [23] extends volumetric convolution to generic point clouds using expansion and restriction operators. PointConv [24] interprets convolution as a Monte Carlo approximation of continuous 3D convolution, and SpiderCNN [25] formulates convolution using step functions and Taylor expansions on k-nearest neighbors. PointCNN [26] mitigates shape degradation and point-ordering bias via X-Conv transformation, and ConvPoint [27] adopts continuous kernels instead of discrete grids. To improve robustness, RIConv [28] exploits low-level rotation-invariant geometric features, and viewpoint-invariant coordinate transformations are used to strengthen sampling stability across different local regions.

Since point clouds occupy non-Euclidean space, graph-based methods provide a natural way to model spatial relations by constructing topological connections between points. As a pioneering approach, Edge-conditional Convolution (ECC) [29] builds learnable filters on graph edges. DGCNN [30] dynamically updates graphs in the feature space to support adaptive relation learning, and LDGCNN [31] simplifies the architecture while enhancing multi-level feature fusion. GAPointNet [32] embeds graph attention into MLPs for efficient local feature extraction, M-GCN [33] captures multi-scale structural patterns using multi-scale graph convolution, and EDGCNet [34] integrates graph convolution into an encoder–decoder framework to enhance efficiency and contextual relation.

Long-range dependencies are difficult to capture using conventional convolution or graph convolution. By comparison, attention-based networks feature weight adaptivity and offer strong global modeling capabilities. The Point Transformer introduces the Transformer structure into point cloud learning and uses local attention to simulate convolutional receptive fields. PCT [35] enhances efficiency and representation ability using offset attention and normalization. PTv2 [36] and PTv3 [37] further reduce computational costs using grouped vector attention and sequential neighborhood mapping. In addition, GDANet [38] improves geometric awareness through disentangled complementary attention, GBNet [39] and SCA-Net [40] enhance spatial and channel-wise fusion, and PointConT [41], SparseFormer [42], and LG-Net [43] balance expressiveness and efficiency through clustering, sparsification, and long-short range modeling. Thanks to their powerful global modeling ability, attention-based approaches consistently achieve state-of-the-art results in classification, part segmentation, and semantic segmentation.

As simple yet effective building blocks, MLPs are widely used for point cloud feature learning. PointNet [11] pioneered end-to-end point cloud learning by achieving transformation invariance and order invariance through T-Net and max pooling. PointNet++ [12] extends it into a hierarchical feature extractor, and PointNeXt [44] further improves performance through optimized training strategies and modernized designs. PointMixer [45] adapts the MLP-Mixer structure for point cloud understanding, and PointMLP [46] uses lightweight geometric affine modules to adaptively refine local features. SO-Net [47] exploits self-organizing maps for hierarchical feature abstraction, PointWeb enhances local context using adaptive feature adjustment, and PointASNL [48] reduces sensitivity to noise and outliers using adaptive sampling and correction.

To intuitively compare the performances of existing point cloud classification and segmentation methods, Table 1 summarizes several aforementioned representative approaches in terms of overall accuracy (OA), parameter count, and computational complexity (GFLOPs) on the ModelNet40 dataset. All experimental results reported in the table are retrieved from published peer-reviewed works. As observed, the proposed AFE-PointNet achieves an OA of 93.6% with only 0.92 M parameters and 8.60 GFLOPs, achieving a favorable trade-off between classification accuracy, model compactness, and computational efficiency. Consequently, AFE-PointNet not only delivers competitive classification performance but also exhibits superior lightweight characteristics, making it highly suitable for practical deployment scenarios with limited computational resources.

## 3. Proposed Methods

**Figure 1:**
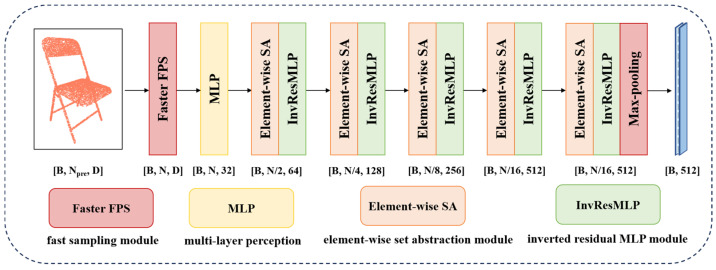
Overall architecture of the AFE-PointNet backbone network.

The overall architecture of the AFE-PointNet backbone network is shown in Figure 1. It consists of a sampling layer, an initial feature mapping MLP, and multiple feature extraction modules. First, a fast-sampling module (Faster FPS) based on bucket partitioning and candidate point selection (QuickFPS [49]) is used to achieve efficient downsampling, reducing computational complexity while ensuring sampling uniformity. Then, an MLP is used for initial feature encoding to increase the number of feature channels and enhance the overall generalization ability and robustness of the model. In the feature extraction stage, a cascaded structure of an element-wise set abstraction module (Element-wise SA) and an InvResMLP is adopted to extract deep multi-scale features from local point cloud neighborhoods. Finally, to further enhance deep feature representation, the network incorporates an additional set of Element-wise SA and InvResMLP modules to perform feature refinement while keeping the number of points and feature dimensions unchanged, and aggregates global features through a max-pooling layer.

### 3.1. Element-Wise Set Abstraction Module (Element-Wise SA)

**Figure 2:**
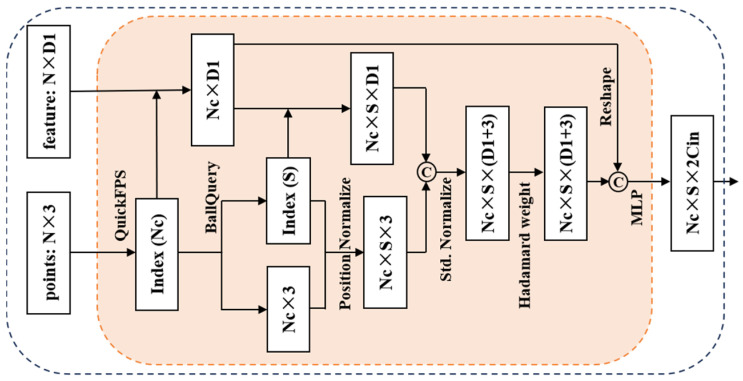
Architecture of the element-wise SA module.

The element-wise weighted set abstraction module is the core component proposed in this paper for adaptive extraction and aggregation of local point cloud features. It replaces the standard SA block in traditional PointNet++ to precisely characterize local geometric topological relationships and boost feature representation performance. The data dimension changes and computational pipeline at each stage of this module are visualized in Figure 2.

Specifically, the raw input point cloud consists of N points, where each point is equipped with a 3D spatial coordinate and a $D_{1}$ dimensional raw feature. Accordingly, the input coordinate tensor and feature tensor are formatted as N × 3 and $N\;\times \;D_{1}$, respectively. First, the QuickFPS downsampling algorithm is adopted to select Nc key points from the total N input points with $N_{c}\;\ll \;N$, yielding key point coordinates of size $N_{c}\;\times \;3$ and corresponding key point features of size $N_{c}\;\times \;D_{1}$. Afterwards, the BallQuery operation retrieves S neighboring points for each sampled key point, constructing neighborhood coordinates with dimension $N_{c}\;\times \;S\;\times \;3$ and neighborhood features with dimension $N_{c}\;\times \;{S\times D}_{1}$.

The obtained neighborhood coordinates are position-normalized relative to their corresponding key points to eliminate absolute positional offset, while the neighborhood features undergo standard normalization to alleviate discrepancies in feature distribution. The normalized coordinates and features are concatenated along the feature dimension to generate fused combined features of $N_{c}\;\times \;S\;\times \;\left(D_{1}+3\right)$. These combined features are fed into an MLP to produce an element-wise weight matrix sharing the identical dimension $N_{c}\;\times \;S\;\times \;\left(D_{1}+3\right)$. A Hadamard product is implemented between the generated weight matrix and concatenated features to adaptively strengthen valid local geometric cues and suppress redundant useless information. Ultimately, after the Reshape operation and a successive MLP transformation, the module outputs aggregated features with a dimension of $N_{c}\;\times \;S\;\times \;2C_{\mathrm{in}}$, where $C_{\mathrm{in}}$ denotes the base input channel number of the subsequent mapping MLP, and the factor of 2 means the final feature channels are doubled after nonlinear transformation.

For a single point $p_{i}$ in a point cloud $P=\left\{p_{1},\;p_{2},\;\ldots ,\;p_{n}\right\}$, assuming its neighboring points in the neighborhood $N_{k}\left(p_{i}\right)$ are $q_{j}\;(1\leq j\leq k)$, with $N_{k}\left(p_{i}\right)$ as input, the calculation method of the element-wise weighting unit is as follows:

(1) $$q_{j}^{\prime }=\mathrm{Norm}\left(\mathrm{MLP}\left(\left[q_{j}-p_{i},\;q_{j}\right]\right)\right)⊙\mathrm{MLP}\left(\left[q_{j}-p_{i},\;q_{j}\right]\right)$$

where Norm denotes the normalization unit, which includes two stages: relative position normalization and standard deviation normalization. The specific calculation formula of the Norm normalization unit is:

(2) $$Norm\left(q_{j}\right)=\frac{q_{j}-p_{i}}{\sigma +\varepsilon }$$

where ε is a very small constant and σ is the normalized standard deviation. For point cloud data with uniform data scale (containing only coordinate features), a global normalized standard deviation $\sigma_{global}$ is used to ensure a uniform distribution of the overall point cloud data. For point cloud data containing multi-dimensional information such as color, normal vectors, and curvature, different feature channels often have different physical magnitudes. Therefore, a channel-wise normalized standard deviation $\sigma_{channel}$ is used to maintain the independence of each channel’s information and prevent some channels from dominating the overall distribution due to excessively large or small scales. The calculation formulas for $\sigma_{global}$ and $\sigma_{channel}$ are as follows:

(3) $$\sigma_{global}=\sqrt{\left(\frac{1}{N\times S\times D}\right)\sum_{i=1}^{N}\sum_{j=1}^{S}{\left(q_{j}-p_{i}\right)}^{2}}$$

(4) $$\sigma_{channel}=\sqrt{\left(\frac{1}{N\times S}\right)\sum_{i=1}^{N}\sum_{j=1}^{S}{\left(q_{j}-p_{i}\right)}^{2}}$$

where N denotes the number of key points after downsampling, S is the number of neighboring points per key point obtained via ball query, D is the feature dimension of each point.

During local feature aggregation, translation invariance means that the network’s performance should be consistent regardless of how the point cloud moves in space. Since the relative coordinates remain unchanged after the reference point moves synchronously with translation, the influence of translation can be eliminated by representing point coordinates as relative positions with respect to the centroid or local reference point. For the neighborhood center point, the query radius used in the ball query process is typically less than 1, and the value of the relative position is smaller than the query radius, which increases the convergence difficulty of the weight matrix $W_{mlp}$ during the Hadamard product weighting. Therefore, this paper normalizes the relative coordinates by:

(5) $$∆q_{j}=\frac{\left(q_{j}-p_{i}\right)}{r}$$

where *r* is the query radius used in the ball query. Relative coordinate normalization not only reduces the training difficulty of the model but also helps to reduce the standard deviation of relative coordinates between feature extraction layers with different query radii in the cascaded structure.

In addition to transformations such as rotation and translation, real point cloud data may suffer from affine transformations and scaling due to camera distortion during acquisition. The Element-wise SA module mitigates the impact of uniform scaling through standard deviation normalization, i.e., dividing the relative coordinates $∆q_{j}$ by the standard deviation σ. After standardization, the data distribution approaches zero mean and unit variance, making the model more robust to scale changes. Furthermore, standardization facilitates model training convergence and reduces training instability caused by data at different scales.

The Hadamard product is often used for element-wise operations between attention weights and feature maps, gating mechanisms, and gradient update rule design. It is defined as the direct product of corresponding elements of two matrices of the same dimension. For matrices A and B of dimension m×n, each element in the resulting matrix H of the Hadamard product satisfies $H_{ij}={\left(A⊙B\right)}_{ij}=A_{ij}\times B_{ij}$, where ⊙ is the symbol for the Hadamard product. Taking a 3×3 matrix as an example:

(6) $$A⊙B=\left[\begin{array}{ccc} a_{11} & a_{12} & a_{13}\\ a_{21} & a_{22} & a_{23}\\ a_{31} & a_{32} & a_{33} \end{array}\right]⊙\left[\begin{array}{ccc} b_{11} & b_{12} & b_{13}\\ b_{21} & b_{22} & b_{23}\\ b_{31} & b_{32} & b_{33} \end{array}\right]=\left[\begin{array}{ccc} a_{11}b_{11} & a_{12}b_{12} & a_{13}b_{13}\\ a_{21}b_{21} & a_{22}b_{22} & a_{23}b_{23}\\ a_{31}b_{31} & a_{32}b_{32} & a_{33}b_{33} \end{array}\right]$$

This operation requires the input matrices to have the same shape. Unlike standard matrix multiplication, which targets linear combinations of rows and columns, the Hadamard product achieves element-wise enhancement or suppression of feature matrices through multiplication operations on local elements. An element-wise feature weighting unit is constructed based on the Hadamard product to automatically learn the importance weights of each dimension of features through end-to-end learning, thereby achieving enhancement of geometric information and suppression of redundant information.

### 3.2. Inverted Residual Multi-Layer Perceptron Module (InvResMLP)

**Figure 3:**
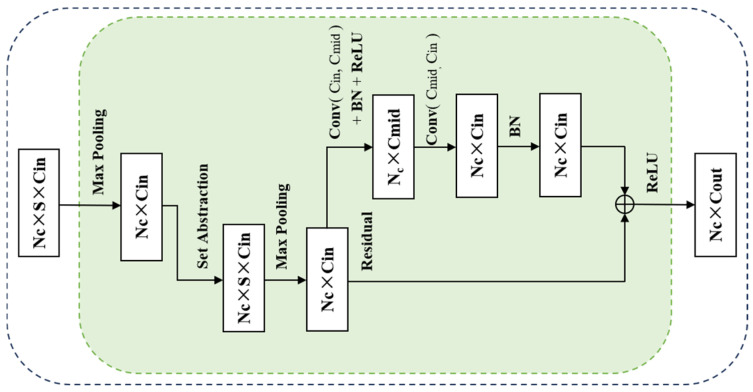
Architecture of the InvResMLP module.

The element-wise weighted SA module yields discriminative local geometric features and provides high-quality geometric representations for subsequent deep feature extraction by InvResMLP. The cascade of these two modules forms a complete pipeline of fine-grained local modeling plus efficient deep feature aggregation, improving prediction accuracy and robustness on point cloud classification and part segmentation tasks. Derived from the inverted residual bottleneck structure of MobileNetV2 [50], InvResMLP is a lightweight quantized feature extraction module, and its detailed computation flow and per-stage dimension transformation are visualized in Figure 3.

The InvResMLP module takes neighborhood features with dimension $Nc\times S\times C_{in}$ as input, achieves deep feature extraction through two parallel feature processing paths, and uses residual connections to ensure gradient stability, ultimately outputting enhanced features of key points. Specifically, the input feature tensor of size $Nc\times S\times C_{in}$ is compressed into $Nc\times C_{in}$ through max pooling. The compressed tensor is then input into an SA unit without farthest point sampling for point-wise neighborhood information search, and the neighborhood information is input into max pooling for secondary feature aggregation, yielding shortcut aggregated features with dimension $Nc\times C_{\mathrm{in}}$. Afterwards, on the main residual branch, the pooled feature $Nc\times C_{\mathrm{in}}$ is sequentially processed by stacked network layers. The first $\mathrm{Conv}\left(C_{\mathrm{in}}{,C}_{\mathrm{mid}}\right)+\mathrm{BN}+ReLU$ expands feature channels from $C_{\mathrm{in}}$ to $C_{\mathrm{mid}}$ to enable high-dimensional feature learning, while the subsequent $\mathrm{Conv}\left({C_{\mathrm{mid}},C}_{\mathrm{in}}\right)$ plus BN reduces channels back to $C_{\mathrm{in}}$ and outputs main-path features of size $Nc\times C_{\mathrm{in}}$. Element-wise summation is then performed between main-branch features and shortcut aggregated features, followed by ReLU activation to generate final enhanced key point features with dimension $Nc\times C_{\mathrm{out}}$, where $C_{\mathrm{out}}$ denotes the number of output feature channels of the InvResMLP block. Benefiting from residual connections, InvResMLP achieves sufficient local feature mining under lightweight calculation overhead while ensuring stable model convergence.

### 3.3. AFE-PointNet Classification Network

**Figure 4:**
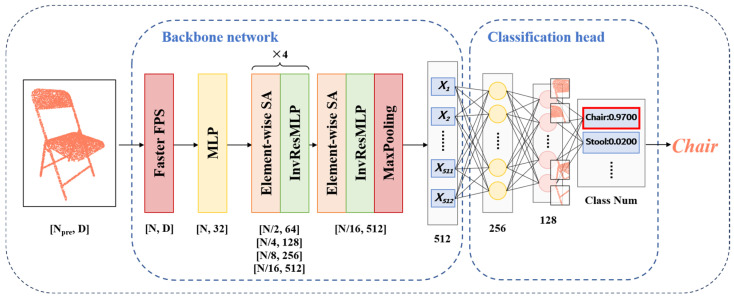
Architecture of the AFE-PointNet network for point cloud classification.

The architecture of the AFE-PointNet network for point cloud classification is shown in Figure 4. It consists of two main parts: the backbone network and the classification head. The backbone network takes the raw point cloud as input. First, efficient downsampling is performed through the Faster FPS module, followed by initial feature encoding through an MLP. Then, a cascaded structure of four groups of Element-wise SA and InvResMLP is used to achieve multi-scale feature extraction and progressive downsampling. Finally, global feature vectors are aggregated through max pooling. The classification head uses two fully connected layers to perform nonlinear transformation on the global features and ultimately outputs the predicted probabilities of each object category, completing the point cloud classification task.

### 3.4. AFE-PointNet Segmentation Network

**Figure 5:**
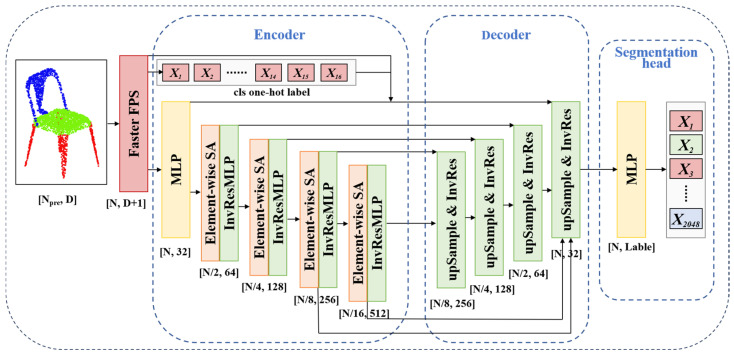
Architecture of the AFE-PointNet network for point cloud segmentation.

The architecture of the AFE-PointNet network for point cloud segmentation is shown in Figure 5. It adopts an encoder–decoder structure to achieve multi-scale feature extraction and fusion. First, efficient downsampling is performed through the Faster FPS module, and one-hot category labels are introduced as conditional information. Then, the encoder achieves multi-scale feature extraction and progressive downsampling through an MLP and four groups of cascaded Element-wise SA and InvResMLP, and passes features from each layer to the decoder via skip connections. The decoder uses feature upsampling modules (upSample and InvRes) to gradually upsample and fuse multi-scale features, ultimately restoring point-wise features. The segmentation head transforms the features through an MLP layer and outputs part category prediction results, completing the point cloud part segmentation task.

## 4. Experiments

### 4.1. Datasets

To verify the classification and segmentation performance of the proposed algorithm, classification comparison experiments were conducted on the ModelNet40 [14] and ScanObjectNN [51] datasets, and segmentation comparison experiments were conducted on the ShapeNetPart [52] dataset.

(1) ModelNet40: A standard synthetic point cloud classification dataset covering 40 categories, containing 12,311 models, of which 9843 are used for training and 2468 for testing (each sample is downsampled to 1024 points).
(2) ScanObjectNN: A real-scene point cloud classification dataset collected by a 3D scanner, containing 15 categories of real objects with severe noise and density variations. It has 2880 training samples and 1440 testing samples (each sample is downsampled to 1024 points).
(3) ShapeNetPart: A point cloud part segmentation dataset containing 16 categories of 3D models, with a total of 14,074 training samples and 2874 testing samples (each sample is downsampled to 2048 points), with a total of 50 part segmentation labels.

**Figure 6:**
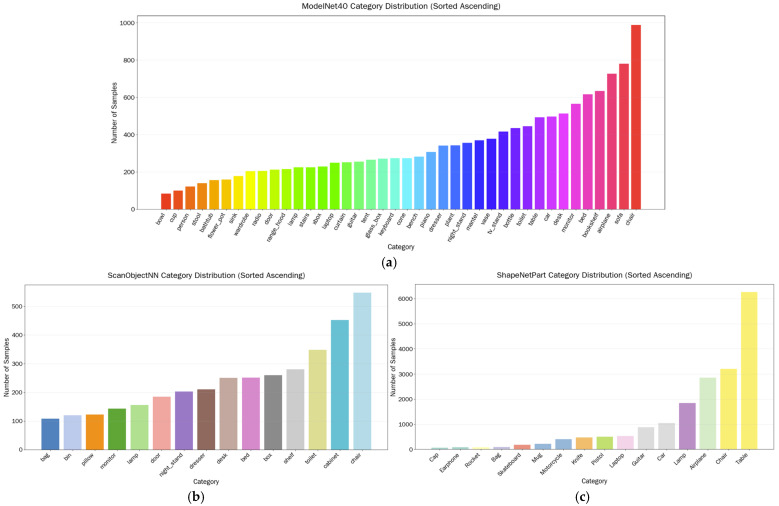
The histograms of sample distribution across all categories for the three datasets (**a**) ModelNet40; (**b**) ScanObjectNN; and (**c**) ShapeNetPart.

Figure 6 illustrates the histograms of sample distribution across all categories for the three datasets. As observed from the histograms, ScanObjectNN has a relatively balanced category distribution with slight numerical discrepancies among classes, where only a few categories such as chair and cabinet contain relatively more samples. ModelNet40 follows an obvious long-tailed distribution: categories like bowl and cup have scarce samples, while chair and airplane are dominated by far more instances, leading to poor overall balance. ShapeNetPart suffers the most severe class imbalance, showing extreme polarization with abundant samples for large objects including table, chair, and airplane and extremely limited data for small accessories such as cap, earphone, and bag.

### 4.2. Evaluation Metrics

Overall accuracy (OA) and class mean accuracy (mACC) are used to evaluate the classification performance. OA represents the overall classification accuracy across all samples, while mACC denotes the average classification accuracy for each category. Instance mean intersection over union (Ins.mIoU) and class mean intersection over union (Cls.mIoU) are adopted as the evaluation metrics for segmentation performance. Ins.mIoU is defined as the average IoU across all point cloud instances, while Cls.mIoU is defined as the average IoU across all object categories. IoU measures the overlap ratio between the predicted segmentation and the ground truth. Their formulas are given as follows:

(7) $$\mathrm{OA}=\frac{\mathrm{TP}+\mathrm{TN}}{\mathrm{TP}+\mathrm{TN}+\mathrm{FP}+\mathrm{FN}}$$

(8) $$\mathrm{mACC}=\frac{1}{C}\sum_{j=1}^{C}\frac{{\mathrm{TP}}_{j}}{{\mathrm{TP}}_{j}+{\mathrm{FN}}_{j}}$$

(9) $$\mathrm{IoU}=\frac{\mathrm{TP}}{\mathrm{TP}+\mathrm{FP}+\mathrm{FN}}$$

(10) $$\mathrm{Ins}.\mathrm{mIoU}=\frac{1}{N}\sum_{i=1}^{N}{\mathrm{IoU}}_{i}$$

(11) $$\mathrm{Cls}.\mathrm{mIoU}=\frac{1}{C}\sum_{j=1}^{C}{\mathrm{IoU}}_{j}$$

where TP, TN, FP, and FN denote the numbers of true positives, true negatives, false positives, and false negatives, respectively, for the overall classification task; ${\mathrm{TP}}_{j}$ and ${\mathrm{FN}}_{j}$ represent the true positives and false negatives for category j, respectively; N is the total number of point cloud instances; C is the total number of object categories; ${\mathrm{IoU}}_{i}$ is the IoU of the i-th instance; and ${\mathrm{IoU}}_{j}$ is the IoU of category j.

### 4.3. Comparison Experiments

In the experiments, we compared the proposed AFE-PointNet with 14 recent state-of-the-art methods, which can be categorized into four groups: point-based hierarchical networks (PointNet [11], PointNet++ [12], SO-Net [47], SoftPoolNet [53], and DRNet [54]); graph-based and relation-aware networks (DGCNN [30], GAPointNet [32], and PRA-Net [55]); point convolution and transformation networks (SpiderCNN [25], PCNN [23], ConvPoint [27], and PointCNN [26]); and spatial partition and multi-view-based networks (Kd-Net [16] and MVTN [21]).

The experimental platform was equipped with a 12th Gen Intel(R) Core (TM) i5-12400F processor, 32 GB of RAM, and an NVIDIA GeForce RTX 2080 Ti graphics adapter. It was based on the Windows 11 operating system, used the PyTorch 2.1.0 deep learning framework, and was configured with the corresponding version of CUDA 11.6. The programming language was Python 3.10.

#### 4.3.1. Classification Experiments on ModelNet40

Table 2 shows the comparison of the classification performances of different methods on the ModelNet40 dataset. The results show that the proposed AFE-PointNet achieves a mACC of 91.2% and an OA of 93.6%, outperforming all compared methods. Compared with PointNet++, AFE-PointNet improves OA by 1.7 percentage points and mACC by 2.8 percentage points. Compared with improved models such as DGCNN, PCNN, and PointCNN, its OA is 0.7, 1.3, and 1.6 percentage points higher, respectively, achieving higher classification accuracy. In terms of mACC, AFE-PointNet reaches 91.2%, outperforming DGCNN (90.2%), SoftpoolNet (89.8%), and GAPointNet (89.7%) by 1.0, 1.4, and 1.5 percentage points, respectively. The comparison experiments show that AFE-PointNet not only surpasses traditional PointNet series methods but also significantly outperforms mainstream improved models based on convolution, graph convolution, and pooling on the ModelNet40 dataset, verifying its effectiveness and superiority in 3D object classification tasks. This performance improvement is mainly attributed to two design advantages. First, the element-wise weighted set abstraction module based on the Hadamard product, which can adaptively enhance key local geometric features and effectively capture fine-grained structural information of objects. Second, the inverted residual MLP module enables efficient deep feature mining in a high-dimensional space while ensuring training stability through residual connections.

**Figure 7:**
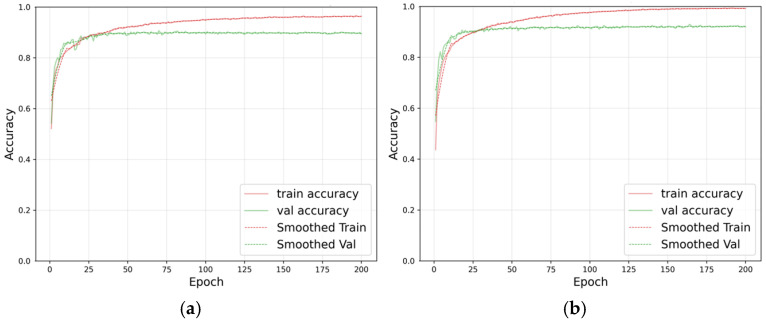
Training and validation accuracy curves of AFE-PointNet and PointNet++ on the ModelNet40 dataset: (**a**) PointNet++; (**b**) AFE-PointNet.

Figure 7 shows the training–validation accuracy curves of PointNet++ and the proposed AFE-PointNet on the ModelNet40 dataset. Both models exhibit typical convergence behavior, with accuracy rising rapidly before plateauing. However, substantial differences are observed in convergence speed and final accuracy. In terms of convergence speed, PointNet++ requires more than 50 epochs to gradually reach a validation accuracy of approximately 92%, and its validation curve continues to fluctuate throughout training. In contrast, AFE-PointNet achieves over 95% validation accuracy within only 25 epochs and stabilizes quickly thereafter, demonstrating significantly improved convergence efficiency. This is attributed to the discriminative local features provided by the element-wise weighted set abstraction module and the optimized gradient propagation path of the inverted residual MLP. Regarding final performance, PointNet++ reaches a peak validation accuracy of around 92–93%, while AFE-PointNet consistently maintains an approximately 99% validation accuracy with notably smaller fluctuations. Both models maintain a relatively small gap between training and validation accuracy throughout training, indicating limited overfitting. The lightweight design of AFE-PointNet reduces parameter redundancy, and the multi-scale feature fusion mechanism enhances feature robustness, contributing to its superior accuracy and stability.

#### 4.3.2. Classification Experiments on ScanObjectNN

The ScanObjectNN dataset is based on point cloud data from real scenes, containing complex interferences such as background noise, uneven point density, and geometric deformations, imposing higher requirements on the robustness and generalization ability of models. Table 3 shows the comparison of classification performance of different methods on this dataset. The experimental results show that the proposed AFE-PointNet achieves a mACC of 82.8% and an OA of 84.5%, reaching the optimal level among all compared methods. Compared with PointNet++, AFE-PointNet improves mACC by 13.0 percentage points and OA by 10.8 percentage points. Compared with representative methods such as DGCNN, PointCNN, DRNet, and MVTN, its OA is 6.4, 6.0, 4.2, and 1.7 percentage points higher, respectively, demonstrating significant performance advantages. Compared with the improvements on ModelNet40, the performance gain of AFE-PointNet on ScanObjectNN is even more prominent, fully verifying the robustness advantage of AFE-PointNet in complex real-world scenes.

**Figure 8:**
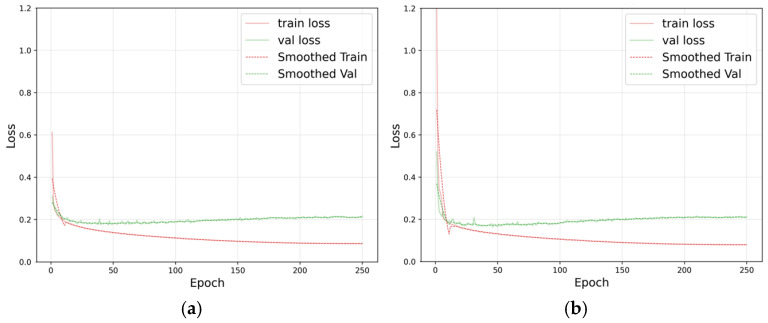
Training and validation loss curves of AFE-PointNet and PointNet++ on the ScanObjectNN dataset: (**a**) PointNet++; (**b**) AFE-PointNet.

Figure 8 compares the training and validation loss curves of PointNet++ and AFE-PointNet on the ScanObjectNN dataset. Both models show decreasing loss and convergence as training progresses, but significant differences are observed in convergence speed, final loss values, and overfitting control. In terms of convergence speed, PointNet++ exhibits relatively slow loss reduction. Its training loss gradually decreases to approximately 0.1, while its validation loss remains stable at around 0.18 throughout training, resulting in a persistent gap between training and validation losses. In contrast, AFE-PointNet achieves rapid loss reduction within approximately 50 epochs. Its training loss quickly converges to 0.1, and its validation loss steadily decreases from 0.34 to approximately 0.1, demonstrating substantially improved convergence efficiency. Regarding overfitting control, PointNet++ shows a consistently large gap between training and validation losses (approximately 0.17) throughout training, indicating limited generalization ability. By contrast, AFE-PointNet maintains a consistently small gap between training and validation losses, with both curves converging to near-zero values and showing no upward trend. This demonstrates that AFE-PointNet effectively suppresses overfitting and achieves stronger generalization ability.

#### 4.3.3. Part Segmentation Experiments on ShapeNetPart

Table 4 presents the part segmentation performance of different methods on the ShapeNetPart dataset. The proposed AFE-PointNet achieves an Ins.mIoU of 86.7% and a Cls.mIoU of 83.6%, which surpasses all comparative approaches. Specifically, our method improves Ins.mIoU and Cls.mIoU by 1.6% and 1.8% over PointNet++, and outperforms DGCNN, PCNN, and SpiderCNN by 1.5%, 1.0; 586%, and 1.4% in terms of Ins.mIoU, respectively.

AFE-PointNet achieves state-of-the-art results across various challenging categories, including backpack (83.6%), car (78.9%), chair (91.3%), earphone (77.2%), knife (88.1%), laptop (95.9%), motorcycle (73.8%), mug (95.1%), and table (82.7%). It demonstrates superior capability in processing objects with complex geometries, ambiguous boundaries, and intricate local details. For representative categories such as chair and mug, our method reaches 91.3% and 95.1% IoU, substantially exceeding other competitors. It also achieves prominent gains on structurally difficult objects like motorcycle and earphone, validating its strong robustness to diverse geometric variations. These consistent performance benefits are attributed to the synergy of three core designs. The Hadamard-product-based element-wise weighted set abstraction enhances feature discrimination. The inverted residual MLP enables deep feature learning while alleviating overfitting. Furthermore, the encoder–decoder structure with skip connections and multi-scale upsampling effectively fuses multi-level features to preserve local details and reconstruct precise part boundaries.

Nevertheless, per-class evaluation reveals minor performance gaps on several individual categories. Our method obtains 83.9% IoU on airplane, slightly lower than DGCNN (84.0%), since graph convolutions better capture long-range structural dependencies between wings and tails. AFE-PointNet achieves 85.6% on cap, which is inferior to SO-Net (88.0%) due to limited training samples and severe intra-class shape variations. It obtains 91.2% on guitar, comparable to PointNet (91.5%), owing to its simple and regular topology. For lamp, our IoU of 84.2% is marginally lower than PCNN (85.0%), as dense convolutions are more adept at modeling delicate and sparse structures. The pistol result of 82.7% is comparable to SpiderCNN and slightly weaker than PCNN (83.3%). For the slender rocket, our 62.7% IoU lags behind DGCNN (63.5%), as dynamic graph learning favors elongated shapes. In addition, our 76.2% accuracy on skateboard is close to PointNet++ (76.4%), indicating that ultra-thin structures remain a common challenge for existing point cloud segmentation methods.

**Figure 9:**
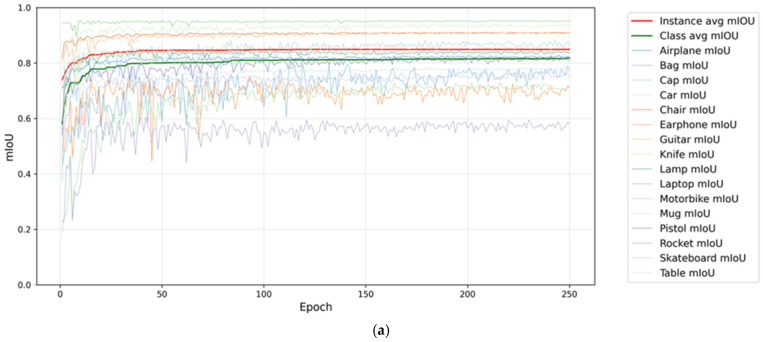

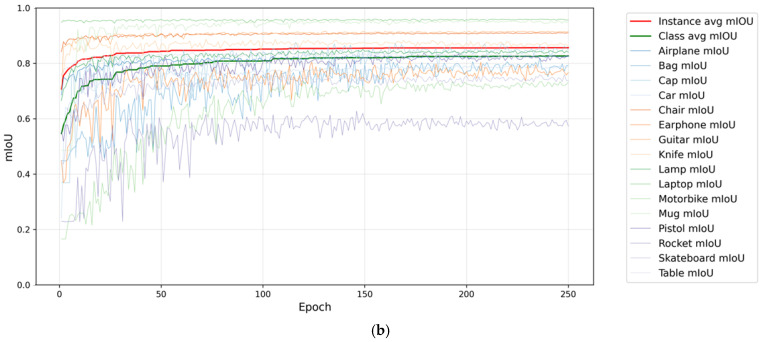
mIoU Curves of AFE-PointNet and PointNet++ on the ShapeNetPart dataset: (**a**) PointNet++; (**b**) AFE-PointNet.

Figure 9 compares the training mIoU curves of PointNet++ and the proposed AFE-PointNet on the ShapeNetPart dataset, including instance average mIoU and class average mIoU as training progresses. From the overall trend, both models exhibit an overall upward trend, with mIoU increasing from approximately 0.74 to higher values. However, substantial differences are observed in convergence speed, data completeness, and final performance. In terms of convergence behavior, PointNet++ shows relatively slow and uneven progress, with its instance mIoU reaching approximately 0.93 by epoch 100. Notably, the training data for PointNet++ contain numerous missing entries across multiple categories (e.g., bag, cap, car, etc.), indicating potential instability or incomplete recording during training. In contrast, AFE-PointNet demonstrates smooth and consistent convergence throughout the entire training process. Its instance mIoU rises steadily from 0.74 to 0.95 by epoch 120, with complete and clean data across all categories, demonstrating significantly improved convergence efficiency and training stability. Regarding final performance, AFE-PointNet achieves an instance mIoU of 0.95 and a class mIoU of 0.95 by epoch 120, outperforming PointNet++ (approximately 0.93). The smooth and complete training curves of AFE-PointNet indicate that the proposed adaptive feature enhancement module effectively accelerates learning, ensures training stability, and achieves superior segmentation accuracy.

## 5. Conclusions

To address the common problems of existing point cloud classification and segmentation methods, such as insufficient local geometric feature extraction, low computational efficiency, and weak robustness to noise and density-uneven data in real scenes, this paper proposes a lightweight point cloud network based on adaptive feature enhancement, named AFE-PointNet. Using PointNet++ as the baseline architecture, the network is improved from three dimensions: feature enhancement, efficient modeling, and computational optimization. First, an element-wise weighted set abstraction module based on the Hadamard product is designed. Through relative coordinate normalization, channel-wise adaptive weighting, and local topology information fusion, precise enhancement of key geometric features is achieved. Second, an inverted residual MLP module is introduced, adopting an inverted bottleneck structure and residual connections to achieve efficient deep feature mining under lightweight constraints. Finally, the Faster FPS fast downsampling strategy is employed, significantly reducing computational cost while ensuring sampling uniformity.

Experimental results on three standard datasets, ModelNet40, ScanObjectNN, and ShapeNetPart, show that AFE-PointNet achieves superior performance in both classification and segmentation tasks compared to mainstream methods such as PointNet++. It demonstrates stronger robustness, especially on real-scene data containing noise and density unevenness, while achieving faster and more stable training convergence. This study verifies the effectiveness of adaptive feature enhancement and lightweight deep mining strategies in point cloud representation tasks. Future work will further optimize the model’s long-range feature modeling capability and explore its applications in 3D object detection, large-scale scene segmentation, and real-time inference on embedded platforms.

## Figures and Tables

**Table 1:** Performance comparison of representative point cloud classification models.

Model	Methods	OA (%)	Params (M)	GFLOPs
PVT [18]	Voxel	94.1	2.76	1.93
CCMNet [19]	Voxel	94.4	3.9	31.8
MVCNN [8]	Multi-view	90.1	11.2	4.37
View-GCN [9]	Multi-view	97.6	23.56	4.42
MVTN [21]	Multi-view	93.5	3.5	1.78
PCNN [23]	Convolution	92.3	8.9	0.29
PointConv [24]	Convolution	92.5	1.8	3.5
PointCNN [26]	Convolution	92.2	0.6	2.3
ECC [29]	Graph	83.2	3.6	7.6
DGCNN [30]	Graph	93.5	1.81	4.8
M-GCN [33]	Graph	93.1	2.1	5.2
PCT [35]	Attention	93.2	2.88	2.32
SparseFormer [42]	Attention	94.2	6.9	20.1
PointNet [11]	MLP	89.2	3.47	12.8
PointNet++ [12]	MLP	91.9	1.48	8.9
PointMLP [46]	MLP	94.1	13.2	31.3
AFE-PointNet	MLP	93.6	0.92	8.6

**Table 2:** Classification results on the ModelNet40 dataset (unit: %).

Methods	mACC	OA
Kd-Net [16]	88.5	91.8
SO-Net [47]	88.5	90.6
PointNet [11]	86.4	90.6
PointNet++ [12]	88.4	91.9
DGCNN [30]	90.2	92.9
PCNN [23]	89.6	92.3
PointCNN [26]	88.2	92.0
SoftpoolNet [53]	89.8	92.3
ConvPoint [27]	88.5	91.8
GAPointNet [32]	89.7	92.4
AFE-PointNet	91.2	93.6

**Table 3:** Classification results on the ScanObjectNN dataset (unit: %).

Methods	mACC	OA
PointNet [11]	63.4	68.2
PointNet++ [12]	69.8	73.7
DGCNN [30]	73.6	78.1
PointCNN [26]	75.1	78.5
DRNet [54]	78.0	80.3
PRA-Net [55]	79.1	82.1
MVTN [21]	80.2	82.8
AFE-PointNet	82.8	84.5

**Table 4:** Part segmentation results on the ShapeNetPart dataset (unit: %).

Methods	Kd-Net [16]	SO-Net [47]	PointNet [11]	PointNet++ [12]	DGCNN [30]	PCNN [23]	SpiderCNN [25]	AFE-PointNet
Ins.mIoU	82.3	84.9	83.7	85.1	85.2	85.1	85.3	86.7
Cls.mIoU	79.8	81.9	80.4	81.8	82.3	81.8	82.4	83.6
IoU								
Airplane	80.1	82.8	83.4	82.4	84.0	82.4	83.5	83.9
Backpack	74.6	77.8	78.7	79.0	83.4	80.1	81.0	83.6
Cap	74.3	88.0	82.5	87.7	86.7	85.5	87.2	85.6
Car	70.3	77.3	74.9	77.3	77.8	79.5	77.5	78.9
Chair	88.6	90.6	89.6	90.8	90.6	90.8	90.7	91.3
Earphone	73.5	73.5	73.0	71.8	74.7	73.2	76.8	77.2
Guitar	90.2	90.7	91.5	91.0	91.2	91.3	91.1	91.2
Knife	87.2	83.9	85.9	85.9	87.5	86.0	87.3	88.1
Lamp	81.0	82.8	80.8	83.7	82.8	85.0	83.3	84.2
Laptop	94.9	94.8	95.3	95.3	95.7	95.7	95.8	95.9
Motorcycle	57.4	69.1	65.2	71.6	66.3	73.2	70.2	73.8
Mug	86.7	94.2	93.0	94.1	94.9	94.8	93.5	95.1
Pistol	78.1	80.9	81.2	81.3	81.1	83.3	82.7	82.7
Rocket	51.8	53.1	57.9	58.7	63.5	51.0	59.7	62.7
Skateboard	69.9	72.9	72.8	76.4	74.5	75.0	75.8	76.2
Table	80.3	83.0	80.6	82.6	82.6	81.8	82.8	82.7

## Data Availability

This study is an experimental analysis of a public dataset. The raw data supporting the conclusions of this article will be made available by the authors on request.
